# Relapse of Neuromyelitis Optica Spectrum Disorder Associated with Intravenous Lidocaine

**DOI:** 10.1155/2011/405837

**Published:** 2011-05-11

**Authors:** Akiyuki Uzawa, Masahiro Mori, Saeko Masuda, Kazuhiko Aoe, Satoshi Kuwabara

**Affiliations:** ^1^Department of Neurology, Graduate School of Medicine, Chiba University, Chiba 260-8670, Japan; ^2^Department of Anaesthesiology, Graduate School of Medicine, Chiba University, Chiba 260-8670, Japan

## Abstract

Lidocaine unmasks silent symptoms and eases neuropathic pain in multiple sclerosis patients; however, the effects of lidocaine in neuromyelitis optica have never been reported. We describe the case of a 59-year-old Japanese woman with neuromyelitis optica spectrum disorder who developed optic neuritis 1 day after intravenous lidocaine injection for treating allodynia. Her symptom seemed to result from a relapse of neuromyelitis optica induced by lidocaine administration, and not because of the transient effects of intravenous lidocaine administration. The possibility that lidocaine administration results in relapse of neuromyelitis optica due to its immunomodulating effects cannot be ruled out.

## 1. Introduction

Lidocaine has been reported to unmask silent symptoms, such as paralysis, hypaesthesia, and visual loss; it has also been shown to abolish painful tonic seizures, neuropathic pain and dysaesthesia in multiple sclerosis (MS) [1]. However, the effects of intravenous lidocaine in neuromyelitis optica (NMO) have never been reported. NMO is an immune-mediated inflammatory disorder of the central nervous system that predominantly affects the optic nerves and spinal cord [2]. Since the discovery of the anti-aquaporin-4 antibody in the sera of patients with NMO [3], several reports have described differences in the pathology [4], immunological status [5], and response to interferon-beta [6] between MS and NMO, indicating that NMO and MS are different diseases. We describe a patient with NMO spectrum disorder who presented with optic neuritis after administration of intravenous lidocaine.

## 2. Case Report

**Figure 1:**
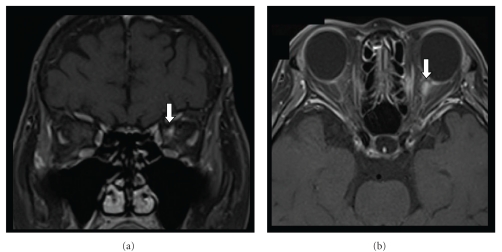
Brain magnetic resonance images with T1-weighted gadolinium enhancement performed 1 week after the onset of left visual loss. A high-intensity lesion on the left optic nerve is seen (white arrows).

A 59-year-old Japanese woman experienced inflammatory episodes with a T1-2 thoracic spinal lesion and a T6-7 thoracic spinal lesion with numbness below the precordia at the age of 57, and a C4-5 cervical spinal lesion and brainstem lesion with diplopia, gait disturbance, and hypalgesia of the right side at age 58. The patients was diagnosed as NMO spectrum disorder because her serum anti-aquaporin-4 antibody titre was positive but not completely fulfilling Wingerchuk's criteria for NMO [2]. The patient suffered from allodynia presenting at the right T2-4 dermatomal area due to a previous thoracic spinal cord lesion and visited an anaesthesiologist. She was then treated with intravenous lidocaine (100 mg) followed by oral mexiletine (150 mg/day) for allodynia. The next day she began to lose left visual acuity, and it gradually worsened. Oral mexiletine was stopped 3 days after treatment began. She consulted us 5 days after she had noted the loss of visual acuity. Neurological examination revealed visual loss in the left eye (visual acuity less than 20/200), and allodynia and hyperalgesia in the right T2-4 dermatomal area. A titre of the anti-aquaporin-4 antibody at that time was higher than that measured 2 and 6 months prior, and lidocaine concentration was below the detection limit (<0.9 pg/mL) in her serum. Brain magnetic resonance images with gadolinium enhancement taken 7 days after the onset of symptom revealed a new left optic nerve lesion (Figure 1). She was diagnosed with optic neuritis and treated with intravenous methylprednisolone (1 g/day) for 3 days and four additional applications of immunoadsorptive plasmapheresis. Her visual loss was completely resolved within a month.

## 3. Discussion

We suspected that the patient's visual loss occurred due to a relapse of NMO, which is presumed to be induced by lidocaine administration. However, the transient effects of intravenous lidocaine administration were ruled out since the patient's visual loss persisted even when her serum lidocaine concentration was below the detection limit and brain magnetic resonance images revealed a new optic nerve lesion. To the best of our knowledge, no studies have reported the relapse of a demyelinating disease that was induced by lidocaine administration. Lidocaine is a sodium channel blocker that unmasks silent symptoms and abolishes neuropathic pain, and dysaesthesia in patients with MS [1], presumably by reducing the size of the action potentials and safety factors for impulse transmission at demyelinated portions of neurons, resulting in the blocking or slowing of nerve conduction [7]. However, the effects of lidocaine are usually only apparent during its administration and are reversible [1] due to the drug's short half-life. We hold some speculations about the association between the administration of lidocaine and relapse of NMO in this case as follows. 

Firstly, lidocaine regulated the immunological conditions of NMO. Some studies have reported that sodium channel blockers can regulate immunological responses, such as promoting Th2-type immune responses [8, 9]. Recently, we had reported that the significant upregulation of Th2- and Th17-related cytokines/chemokines in the cerebrospinal fluid of NMO patients, but not MS patients [5]. In our case, intravenous lidocaine may have modulated the immune system, leading to aggravation of the NMO condition. 

Secondly, lidocaine blocked neural conduction in the unrecognized demyelinated or inflamed sections of neurons in NMO. Lidocaine has been shown to cause irreversible conduction block in vitro [10]. In addition, inflammation causes a decline in the safety factors of conduction. The release of nitric oxide at sites of inflammation is associated with an axonal conduction block; demyelinated axons are particularly sensitive [11]. However, we believed that conduction blocks alone cannot explain the patient's condition because her condition did not worsen immediately after the administration of lidocaine and gadolinium-enhanced brain magnetic resonance images revealed a new optic nerve lesion. 

There are some possible explanations for this case. We cannot deny association between the relapse and administration of intravenous lidocaine was coincidental, but it may be suggested that lidocaine administration led to changes in the patient's immunological condition and resulted in the relapse of NMO. Physicians should recognise that such conditions may occur in patients diagnosed with NMO.

##  Conflict of Interests

The authors declared that there is no conflict of interests.

## References

[1] Sakurai M, Kanazawa I (1999). Positive symptoms in multiple sclerosis: their treatment with sodium channel blockers, lidocaine and mexiletine. *Journal of the Neurological Sciences*.

[2] Wingerchuk DM, Lennon VA, Pittock SJ, Lucchinetti CF, Weinshenker BG (2006). Revised diagnostic criteria for neuromyelitis optica. *Neurology*.

[3] Lennon VA, Kryzer TJ, Pittock SJ, Verkman AS, Hinson SR (2005). IgG marker of optic-spinal multiple sclerosis binds to the aquaporin-4 water channel. *Journal of Experimental Medicine*.

[4] Misu T, Fujihara K, Kakita A (2007). Loss of aquaporin 4 in lesions of neuromyelitis optica: distinction from multiple sclerosis. *Brain*.

[5] Uzawa A, Mori M, Arai K (2010). Cytokine and chemokine profiles in neuromyelitis optica: significance of interleukin-6. *Multiple Sclerosis*.

[6] Uzawa A, Mori M, Hayakawa S, Masuda S, Kuwabara S (2010). Different responses to interferon beta-1b treatment in patients with neuromyelitis optica and multiple sclerosis. *European Journal of Neurology*.

[7] Yokota T, Saito Y, Miyatake T (1994). Conduction slowing without conduction block of compound muscle and nerve action potentials due to sodium channel block. *Journal of the Neurological Sciences*.

[8] Tanaka A, Minoguchi K, Oda N (2002). Inhibitory effect of lidocaine on T cells from patients with allergic asthma. *Journal of Allergy and Clinical Immunology*.

[9] Okada K, Sugiura T, Kuroda E, Tsuji S, Yamashita U (2001). Phenytoin promotes Th2 type immune response in mice. *Clinical and Experimental Immunology*.

[10] Lambert LA, Lambert DH, Strichartz GR (1994). Irreversible conduction block in isolated nerve by high concentrations of local anesthetics. *Anesthesiology*.

[11] Redford EJ, Kapoor R, Smith KJ (1997). Nitric oxide donors reversibly block axonal conduction: demyelinated axons are especially susceptible. *Brain*.

